# Treatment with Myo-Inositol and Selenium Ensures Euthyroidism in Patients with Autoimmune Thyroiditis

**DOI:** 10.1155/2017/2549491

**Published:** 2017-02-15

**Authors:** Maurizio Nordio, Sabrina Basciani

**Affiliations:** Department of Experimental Medicine, University “Sapienza”, Rome, Italy

## Abstract

Clinical evidences have highlighted the efficacy of myo-inositol and selenium in the treatment of autoimmune thyroiditis. Aim of this study was to further analyze the role of myo-inositol plus selenium (Myo-Ins-Se) in restoring a normal thyroid function of Hashimoto's patients with subclinical hypothyroidism. Eighty-six patients with Hashimoto's thyroiditis having thyroid-stimulating hormone (TSH) levels between 3 and 6 mIU/L, elevated serum antithyroid peroxidase (TPOAb) and/or antithyroglobulin (TgAb), and normal free thyroxine (fT_4_) and free triiodothyronine (fT_3_) levels were enrolled in the study: one hyperthyroid subject with TSH about 0.14 *μ*U/ml was included in this trial as a single case. Patients were assigned to receive Myo-Ins-Se. TSH, TPOAb, and TgAb levels were significantly decreased in patients treated with combined Myo-Ins-Se after 6 months of treatment. In addition, a significant fT_3_ and fT_4_ increase, along with an amelioration of their quality of life, was observed. Remarkably, TSH values of the hyperthyroid patient increased from 0.14 *μ*U/ml up to 1.02 *μ*U/ml, showing a complete restoration of TSH values at a normal range. In conclusion, the administration of Myo-Ins-Se is significantly effective in decreasing TSH, TPOAb, and TgAb levels, as well as enhancing thyroid hormones and personal wellbeing, therefore restoring euthyroidism in patients diagnosed with autoimmune thyroiditis.

## 1. Introduction

During the last decades, a sharp increase in thyroid pathology took place in most countries. The reasons for that may be explained not only because we have a better ability to make precocious diagnosis but also because other factors may have contributed to that increase. In this view, genes play an important role, since an individual with a family history positive for thyroid problems has a significantly higher possibility of developing a pathology of the gland. Also, environment may contribute to the development of these pathologies such as radioactive accidents, pollution, and other iatrogenic illnesses, especially those correlated with autoimmunity. For example, in regions with severe selenium (Se) deficiency, a higher incidence of thyroiditis may be documented, due to a decreased activity of selenium-dependent glutathione peroxidase activity within thyroid cells. Selenium-dependent enzymes are also key elements in the regulation of the immune system. Therefore, even mild selenium deficiency may lead to the development and maintenance of autoimmune thyroid diseases. In addition, the so-called “constitutional factors,” such as age and sex, may influence and facilitate the appearance of thyroidal pathologies [1–5]. Among the numerous illnesses, thyroiditis is the most frequent (roughly about 20% of all thyroidal diseases) and is divided as acute, subacute, and chronic. The autoantibodies against thyroid presence are a peculiar feature during the evolution of most of them. A downregulation of suppressor T-lymphocytes and the ensuing activity against thyroglobulin (TgAb) and thyroid-peroxidase (TPOAb), one essential for the production and storage of thyroid hormones and the other involved in hormone synthesis, respectively, appear to be the starting point of the autoimmune process. Once the inflammatory cascade has been activated and the mechanism initiated, T-lymphocytes may trigger a production of specific antibodies by B-lymphocytes [6]. Oxidative stress has been shown to be responsible for the onset of these autoimmunity disorders. Hence, an increase of TPOAb and TgAb concentration is largely seen. Concentration of these antibodies, as well as thyroid morphology, and the ability of follicular cells to produce thyroid hormones may change during life. Anyway, their presence may cause continuous damage to the thyroid tissue, leading to a decrease in hormones production. In fact, in patients with thyroiditis, undergoing long-term follow-up, very often a decline towards hypothyroidism is seen [7].

Inositol is better known as a family of slightly different compounds derived by a C_6_ sugar alcohol. Of the nine 1,2,3,4,5,6-cyclohexanehexol isomers, Myo-Ins is the far most representative, with other inositols (allo-, cis-, d-chiro-, l-chiro-, epi-, muco-, neo-, and scyllo-) being less known, except for d-chiro-inositol that has an important role in insulin signal transduction and insulin resistance [8]. Several studies demonstrated that Myo-Ins is the precursor of the synthesis of phosphoinositides, which are part of the phosphatidylinositol signal transduction pathway across the plasma membrane, via the second messenger 1,4,5-triphosphate that modulates intracellular Ca^2+^ release [9]. Therefore, it acts as a second messenger regulating the activities of several hormones, such as insulin, follicle-stimulating hormone (FSH), and thyroid-stimulating hormone (TSH) [10]. As far as TSH signaling is concerned, after the binding of TSH to its receptor on thyroid cell surface, it stimulates cell growth and differentiation, in addition to thyroid hormone synthesis. This binding with TSH receptors activates two postreceptor cascades: one involves adenylyl cyclase, leading to an increase of intracellular cyclic AMP and protein kinase A phosphorylation and also to an activation of cytosolic and nuclear target proteins; the other is inositol-dependent and involves the phospholipase C-dependent inositol phosphate Ca^2+^/diacylglycerol pathway, resulting in a boost of hydrogen peroxide (H_2_O_2_) generation. In addition, while the cAMP pathway is more involved in cell growth, differentiation, and thyroid hormones (T_4_-T_3_) secretion, the inositol-dependent pathway regulates H_2_O_2_-mediated iodination of thyroglobulin.

TSH, a glycoprotein synthesized and secreted by the pituitary gland, regulates the release of thyroid hormones, triiodothyronine (T_3_) and thyroxine (T_4_), from the thyroid. These hormones modulate many physiological processes in the human cells and are crucial for growth, development, differentiation, and the maintenance of basal metabolism [11]. Most scientists agree in defining the T_4_ more like a “prohormone” rather than a real hormone, although it is the major hormone secreted by the thyroid [12]. T_4_, in fact, is not very active; it expresses the functional activity of the gland, but to be useful to the human body it must be converted to T_3_. Here, through the enzyme 5′ deiodinase, the prohormone is deprived of one iodine and, in this way, is activated. Only T_3_ can enter easily into the tissue cells where it carries out its physiological functions. Hypothyroidism and hyperthyroidism are thyroid disorders that can be caused by TSH signal transduction impairment. Indeed, elevated thyroid-stimulating hormone (TSH) and autoantibodies levels, such as TgAb and TPOAb, are typical features of autoimmune thyroiditis. In such pathologies, among which Hashimoto's thyroiditis (HT) is the most representative, thyroid gland gets underactive, as attacked by cell- and antibody-mediated autoimmune processes [13]. Excluding HT, a mild thyroid failure is called subclinical hypothyroidism (SH) [14] and usually is balanced by a slight TSH level increase, varying in the range 3–6 mIU/L. According to the American Thyroid Association's (ATA's) guidelines, the normal range for TSH values, with an upper limit of 4.12 mIU/L, is largely based on National Health and Nutrition Examination Survey (NHANES) III data, but it has not been universally accepted. In fact, some have proposed that the upper normal TSH values should be either 2.5 or 3.0 mIU/L [15].

The relevant impact of Se on inflammatory activity in thyroid-specific autoimmune disease has already been shown in several trials [16–19], demonstrating its possible therapeutic effect in reducing TPOAb in patients with autoimmune thyroiditis (AIT). AIT like HT, idiopathic myxedema, and Graves' disease are characterized by the high levels of TPOAb (>50 lU/ml), which are closely associated with abnormal levels of TSH (<1.0 mIU/L in hyperthyroidism and >2.5 mIU/L in hypothyroidism forms) and correlated with progressive thyroidal damage and lymphocytic inflammation [20]. Graves' hyperthyroidism and Hashimoto's hypothyroidism might be the opposite spectrums of one disease. In a previous study of ours, the beneficial effect of Myo-Ins in reducing TSH levels through the improvement (increase) of TSH sensitivity was highlighted. Essentially, it was shown that supplementation of Myo-Ins-Se was able to restore the euthyroid state and improve personal wellbeing in subclinical hypothyroidism patients [21].

Taking into account our previous data, the aim of this study was to further investigate the efficacy of Myo-Ins-Se in restoring the euthyroid state of Hashimoto's patients with subclinical hypothyroidism. A single case of hyperthyroidism was also included in the study drawing attention to the unique therapeutic approach of Myo-Ins.

## 2. Patients and Methods

A total of 87 patients, 8 men and 79 women (mean age 42.30 ± 0.06 years), were included in our study; 86 were meeting the inclusion criteria as follows: age range 19–65, TSH levels between 3 and 6 mIU/L, elevated serum TPOAb and/or TgAb, and normal free thyroxine (fT_4_) and free triiodothyronine (fT_3_) levels. A hyperthyroid subject, a woman with TSH around 0.14 mIU/L at baseline, was also selected to enter the study. None of the patients were undergoing adjuvant treatment with trace elements, vitamins, or antidepressive and antipsychotic drugs. Patients were otherwise healthy. Informed consent was obtained from all participants in this study. Patients received tablets containing 600 mg myo-inositol plus 83 *μ*g selenium in the form of L-selenomethionine (Tiroxil®, Lo.Li. Pharma Srl, Rome, Italy) orally for 6 months. Participants were asked to take the medication with water about 2 h before or after meal. Primary outcome was detection of serum TSH levels. Secondary outcomes were TPOAb, TgAb, fT_3_, and fT_4_ hormone concentrations as well as quality of life evaluation.

### 2.1. Laboratory and Technical Investigations

The investigation was performed over a period of 6 months. Blood samples were drawn from each patient, and serum TSH, fT_3_, fT_4_, TPOAb, and TgAb levels were measured at baseline and at the end of the study. TSH, fT_3_, and fT_4_ concentrations were measured by an enzyme immunometric assay (Byk-Sangtec Dietzenbach, Germany). Plasma total TPOAb and TgAb concentrations were measured by a commercial enzyme luminescence assay (Byk-Sangtec, Dietzenbach, Germany). At enrolment and after 6 months, the subjective symptomatology (SS) was evaluated using a questionnaire, including 7 questions testing the presence of symptoms; in particular, the SS comprises local symptoms such as pain localized on the front of the neck, discomfort when swallowing liquids or solids, ability to raise the voice, and feeling “constriction” in the neck wearing high-necked clothes and accessory-type choker necklaces or when lying down [22].

### 2.2. Statistics

Data were processed using paired *t*-tests, with GraphPad Software (GraphPad Software Inc., La Jolla, CA, United States). Values are expressed as median (±SEM), and a *p* value ≤0.05 was utilized throughout as a criterion for any result that was statistically significant.

## 3. Results

**Figure 1:**
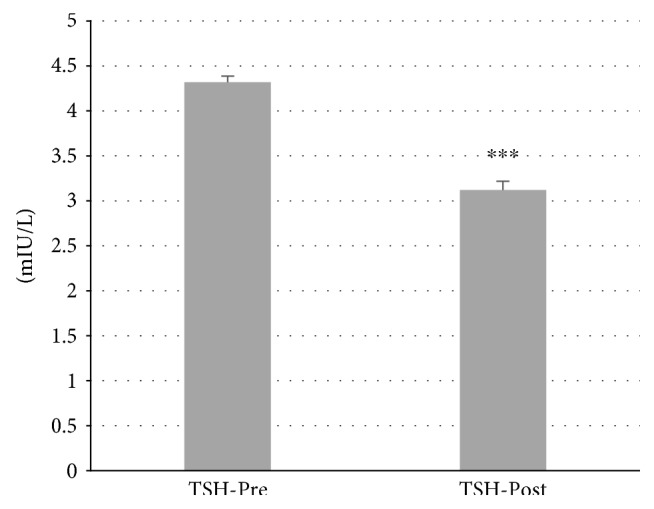
Thyroid-stimulating hormone (TSH) levels in patients at baseline and after 6 months of Myo-Ins-Se treatment (*n* = 86). Values are expressed as median (±SEM). Comparison of TSH levels at baseline versus posttreatment, ^∗∗∗^*p* ≤ 0.001.

**Figure 2:**
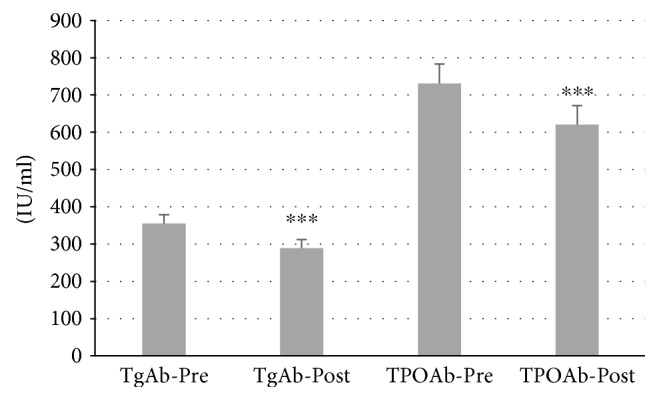
Serum antithyroglobulin (TgAb) and antithyroid peroxidase (TPOAb) levels of patients at baseline and after 6 months of Myo-Ins-Se treatment (*n* = 86). Values are expressed as median (±SEM). Comparison of TgAb and TPOAb levels at baseline versus posttreatment, respectively; ^∗∗∗^*p* ≤ 0.001.

**Figure 3:**
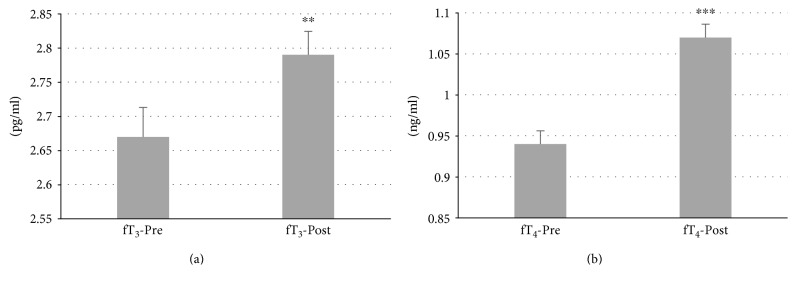
Free triiodothyronine (fT_3_) and free thyroxine (fT_4_) levels of patients at baseline and after 6 months of Myo-Ins-Se treatment (*n* = 86). Values are expressed as median (±SEM). Comparison of fT_3_ (a) and fT_4_ (b) concentration at baseline versus posttreatment, respectively; ^∗∗^*p* ≤ 0.01, ^∗∗∗^*p* ≤ 0.001.

**Figure 4:**
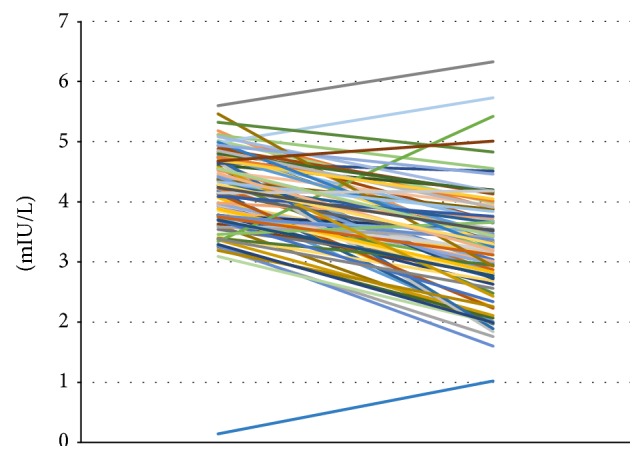
Thyroid-stimulating hormone (TSH) levels in patients at baseline (T0) and after 6 months (T6) of Myo-Ins-Se treatment (*n* = 87). Hyperthyroid patient is included in this graph.

**Figure 5:**
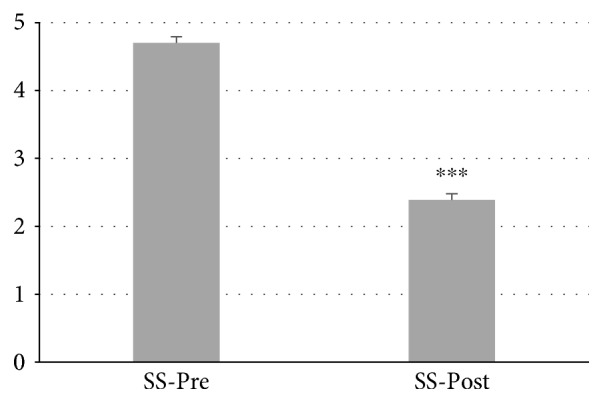
Subjective symptomatology of patients at baseline and after 6 months of Myo-Ins-Se treatment (*n* = 87). Values are expressed as median (±SEM). Comparison of patients' subjective symptomatology at baseline versus posttreatment; ^∗∗∗^*p* ≤ 0.001.

In total, 87 patients with autoimmune thyroiditis were enrolled in the study. The median age of patients was 42.30 ± 0.06 years (range: 19–65). All patients were receiving Myo-Ins-Se treatment for 6 months. A significant reduction of TSH levels was observed in patients' posttreatment, from 4.32 ± 0.06 mIU/L at baseline to 3.12 ± 0.09 mIU/L after treatment (*p* ≤ 0.001) (Figure 1). There were significant decrements in both autoantibodies TgAb and TPOAb serum levels after administration of Myo-Ins-Se: TgAb levels decreased from 344.96 ± 23.77 IU/ml to 288.84 ± 23.35 IU/ml after treatment (*p* ≤ 0.001) and TPOAb from 720.67 ± 52.39 IU/ml to 620.38 ± 50.90 IU/ml, pre- and post-Myo-Ins-Se treatment, respectively (*p* ≤ 0.001) (Figure 2). The serum fT_3_ and fT_4_ levels of patients were slightly but significantly higher at the end of 6-month period when compared with the values at baseline: fT_3_ values were 2.67 ± 0.04 pg/ml at baseline and 2.79 ± 0.03 pg/ml posttreatment (*p* ≤ 0.01) and fT_4_ levels were 0.94 ± 0.02 ng/ml and 1.07 ± 0.02 ng/ml (*p* ≤ 0.001) pre- and posttreatment, respectively (Figure 3). In Figure 4 are shown the TSH values from each patient including also the hyperthyroid patient. The graph clearly shows how TSH levels decreased in Hashimoto's patients with SH but increased in the hyperthyroid patient from T0 to T1 (baseline and 6-month treatment, resp.) (Figure 4). In particular, TSH values of the hyperthyroid patient increased from 0.14 mIU/L up to 1.02 mIU/L, therefore restoring TSH values at a normal range (Figure 4). Questionnaire of SS revealed a significant improvement of patients' quality of life after 6 months of Myo-Ins-Se administration. Values dropped down from 4.67 ± 0.09 at baseline to 2.37 ± 0.09 at the end of treatment (*p* ≤ 0.001) (Figure 5).

## 4. Discussion

The subject of the present study was to further examine the efficacy of Myo-Ins-Se in restoring the euthyroid state in patients affected by thyroid disorders. We could demonstrate that, in patients with AIT, the concentrations of TSH, TPOAb, and TgAb significantly decreased after administration of Myo-Ins-Se for 6 months. Furthermore, quality of life was significantly improved in all patients at the end of this study. A single case of hyperthyroidism was also analyzed, emphasizing the effect of Myo-Ins in increasing TSH levels up to normal concentrations.

Until recent years, the pharmacological approach to inflammatory thyroid pathologies, especially those having a high titer of autoantibodies, was based upon the use of corticosteroids that, of course, are able to temporarily decrease inflammation and antibody concentrations [23, 24]. However, being almost the only way of intervention (FANS are also used), they have been frequently overused, with the consequence of an increase in the percentage of their well-known adverse effects. In this view, a series of compounds able to ameliorate SS, inflammation parameters, and thyroid status have been identified to date. Among them, inositol and Se seem to be the most efficacious in terms of thyroid function recovery and symptomatology amelioration. The story of inositol is a long one, since it started around the last two decades of the previous millennium, when researchers demonstrated its ability as a calcium-mobilizing second messenger [25] and to decrease insulin resistance in polycystic ovary syndrome [26]. Therefore, a widespread research activity was initiated, giving rise to an important series of information, clarifying various aspects of hormonal signal transduction. In particular, it has been shown that relatively low TSH concentrations are able to stimulate cAMP-mediated signal cascade, while only a 100-fold higher TSH concentration is needed to stimulate the inositol-mediated signal cascade [27]. Therefore, it can be speculated that impairment of the inositol-dependent TSH signaling pathway may be, at least in part, one cause of thyroid malfunctioning and that, by increasing the availability of Myo-Ins at cellular level, it is possible to improve TSH sensitivity of the thyroid follicular cell. In fact, previous clinical studies, as well as this one, indicate that the administration of 600 mg Myo-Ins is able to ameliorate thyroid function and symptomatology in patients with HT [21].

The physiological role of TSH is quite crucial in the regulation of hypothalamic-pituitary-thyroid axis, as it modulates the release of the thyroid hormones from the thyroid gland. It prompts iodine uptake by the thyroid [28], induces thyroid epithelial differentiation and growth [29], and preserves thyroid cells from apoptosis [30]. In fact, impairment of TSH signal transduction can lead not only to thyroid disorders such as hypothyroidism and hyperthyroidism but also to proliferation and differentiation of human thyroid carcinoma cells [31]. TSH increase is also associated with a higher risk for coronary heart disease (CHD) accidents, increased CHD mortality, and heart failure (HF) events, particularly in those patients with TSH levels >10.0 mIU/L [32, 33].

In this study, it is shown how Myo-Ins acts on TSH levels lowering them when too high and increasing them if too low. Even lowering the TSH, the production of thyroid hormones, T_4_ and T_3_, is induced. As mentioned above, normally, TSH induces the uptake of iodine by the thyroid gland as well as the production of thyroid hormone; increased levels of TSH stimulate the thyroid to produce more thyroid hormones, thereby returning the level of thyroid hormone in the blood back to normal. Our study shows that, after Myo-Ins-Se supplementation for 6 months, TSH levels significantly decreased in HT's patients with SH without affecting the production of thyroid hormones that, as a matter of fact, were significantly increased after the treatment. Hypothyroidism results from a deficient production of thyroid hormone by the thyroid gland. Since the thyroid hormones regulate metabolism in every cell of the body, a shortage of them can virtually affect all body functions. Deficiency of thyroid hormones can result from lack of stimulation by the pituitary gland, a defective hormone synthesis, or the impaired cellular conversion of T_4_ to T_3_. Another substance widely used in the treatment of AIT is Se. In fact, as reported earlier, it is a trace element, essential to wellbeing, that exerts multiple and complex effects on human health [34]. The physiological functions of Se are carried out by selenocysteine, the 21st amino acid, which is the defining feature of the 25 selenoprotein-encoding genes so far discovered within the human genome [35]. Se can exert an influence on immunological responses, cell growth, and viral defense. It is an essential particle in the active site of enzymes such as glutathione peroxidases, deiodinases, and thioredoxin reductases. In addition, it has a fundamental importance in the synthesis and function of thyroid hormones and protects cells against free radicals and oxidative damage. In fact, Se demonstrates antioxidant and anti-inflammatory properties that have a relevant impact on immune function [36, 37] and it has been shown to reduce the inflammatory status in patients with HT [16]. Intake of Se necessary to maintain suitable selenoenzyme activity is about 55 *μ*g per day. Se deficiency contributes to decreased activity of glutathione peroxidases, which can lead to oxidative damage, or deiodinase, which is connected with impaired thyroid activity. Moreover, a low Se concentration causes autoimmune processes in the thyroid gland; thus Se deficiency is essential to the pathogenesis of autoimmune thyroiditis or Graves' disease. A number of studies have shown indeed the effectiveness of Se in lowering the autoantibodies, in particular the TPOAb concentration in AIT's patients [16, 38, 39]. These findings are in line with our previous [21] and recent results where a significant decrease in serum TPOAb and TgAb levels was observed at the end of Myo-Ins-Se treatment. To be noted, different from the sole supplementation of Se, the combination Myo-Ins-Se is able to decrease not only TPOAb but also TgAb levels.

Therefore, the conclusion of our study is that the supplementation of Myo-Ins-Se is able to restore the euthyroid state as well as improve the wellbeing of Hashimoto's patients with SH. It would be of interest to further investigate the effect of this combined therapy in a more considerable group of hyperthyroid patients since, in this single case, we have obtained very prominent results, showing that Myo-Ins-Se restores TSH levels up to normal values. Bearing in mind also the safety of these two molecules' usage, accentuated by the absence of side effects, the Myo-Ins-Se combination can be considered a very efficacious and safe therapy for AIT treatment.
